# Bacterial Community Analysis of Drinking Water Biofilms in Southern Sweden

**DOI:** 10.1264/jsme2.ME14123

**Published:** 2015-02-21

**Authors:** Katharina Lührig, Björn Canbäck, Catherine J. Paul, Tomas Johansson, Kenneth M. Persson, Peter Rådström

**Affiliations:** 1 Applied Microbiology, Department of Chemistry, Lund UniversityP.O. Box 124, SE-221 00 LundSweden; 2 Sydvatten ABHyllie Stationstorg 21, SE-215 32 MalmöSweden; 3 Microbial Ecology Group, Department of Biology, Lund UniversitySölvegatan 37, SE-223 62 LundSweden; 4 Water Resources Engineering, Department of Building and Environmental TechnologyLund University, P.O. Box 118, SE-221 00 LundSweden

**Keywords:** drinking water, biofilm, next-generation sequencing, bacterial communities, 16S rRNA pyrosequencing

## Abstract

Next-generation sequencing of the V1–V2 and V3 variable regions of the 16S rRNA gene generated a total of 674,116 reads that described six distinct bacterial biofilm communities from both water meters and pipes. A high degree of reproducibility was demonstrated for the experimental and analytical work-flow by analyzing the communities present in parallel water meters, the rare occurrence of biological replicates within a working drinking water distribution system. The communities observed in water meters from households that did not complain about their drinking water were defined by sequences representing *Proteobacteria* (82–87%), with 22–40% of all sequences being classified as *Sphingomonadaceae*. However, a water meter biofilm community from a household with consumer reports of red water and flowing water containing elevated levels of iron and manganese had fewer sequences representing *Proteobacteria* (44%); only 0.6% of all sequences were classified as *Sphingomonadaceae*; and, in contrast to the other water meter communities, markedly more sequences represented *Nitrospira* and *Pedomicrobium*. The biofilm communities in pipes were distinct from those in water meters, and contained sequences that were identified as *Mycobacterium*, *Nocardia*, *Desulfovibrio*, and *Sulfuricurvum*. The approach employed in the present study resolved the bacterial diversity present in these biofilm communities as well as the differences that occurred in biofilms within a single distribution system, and suggests that next-generation sequencing of 16S rRNA amplicons can show changes in bacterial biofilm communities associated with different water qualities.

Biofilms are populations of microorganisms that are typically concentrated at a solid-liquid interface and surrounded by an extracellular polymeric substance matrix (13). The presence of extracellular polymeric substances within the biofilm protects bacteria by making them more resistant to chemicals such as disinfectants (10). The environment within a drinking water distribution system (DWDS) is oligotrophic and can contain disinfectants, with more than 95% of the bacterial biomass occurring as biofilms on the inner surface of the DWDS and less than 5% existing in the planktonic form (9). Biofilms in the distribution system constitute an ecosystem that can influence the esthetic quality of drinking water by altering taste, color, and odor, and also microbial water quality through the detachment of biomass into the bulk water (22). Bacterial biofilms have also been associated with technical problems within the DWDS such as corrosion (41). Although biofilms are known to have these impacts on drinking water, little is known about the mechanisms involved; therefore, a deeper understanding of the mechanisms by which the microbes in this human-built ecosystem participate in the delivery of drinking water to consumers need to be elucidated in more detail.

Less than 1% of bacteria in fresh and drinking water are currently culturable and bacteria in drinking water biofilms can also be present in a viable but non-culturable (VBNC) state (1, 20, 37, 40). Therefore, culture-independent methods are preferable for providing a more complete picture of the microbial community; however, this type of analysis may not distinguish between dead or living cells (30). While methods using clone libraries, DGGE, and other DNA-based methods have contributed descriptions of the microbes present in drinking water biofilms (8, 33, 42), next-generation sequencing (NGS) is considered to provide the most detailed, high throughput, culture-independent method for the characterization of microbial communities. Previous studies that have examined bacterial communities present in water or biofilms of the DWDS have used the NGS of amplicons from water meter biofilms (16), clear well biofilms (44), faucet biofilms (27), and biofilms in a pilot-scale microfiltration plant that treats drinking water (21). NGS has also been used to examine the impact of changing hydraulic regimes on the bacterial biofilm community structure in an experimental distribution system (5) as well as the influence of chloramination and chlorination on planktonic bacterial communities in water samples (19). However, the reproducibility of amplicon NGS for real DWDS biofilms, the diversity present across biofilms from a single distribution system, and the feasibility of using water meters to investigate changes associated with the consumer perception of water quality have not yet been investigated.

We herein examined bacterial communities in DWDS biofilms using a deep 16S rRNA amplicon NGS analysis. To obtain a representative drinking water biofilm, samples from water meters were analyzed from a single existing distribution network in southern Sweden. The reproducibility of this approach was demonstrated by comparing biofilms from water meters installed in parallel and, thus, experiencing nearly identical hydrological environments. In addition, we showed that differences in the biofilm community composition in water meters and pipes as well as perceived water qualities within a single DWDS were resolvable.

## Materials and Methods

### Sampling

Six biofilm samples were collected from the DWDS of the city of Landskrona (Sweden) in March, April, and June, 2011 (Table 1). Water meter biofilms were sampled using sterile cotton transport swabs. Biofilm samples from the walls of the pipes were taken with a sterile cell spatula (TPP, Trasadingen, Switzerland) and the collected material was resuspended in approximately 5 mL of water from the sampling site. All samples were transported to the laboratory in a cooling box within 8 h after sampling and then stored at −20°C until further analyses. Water samples for water quality testing were taken from households during the sampling of water meter biofilms and the results obtained were reported together with general water quality data representative for drinking water in the city of Landskrona (Sweden) (Table 2). Water quality testing was performed by ALcontrol AB, Malmö, Sweden using standard methods (pH: SS028122-2; conductivity: SS-EN 27888-1; hardness: calculated from magnesium and calcium concentrations; calcium: SS-EN ISO 11885-1; magnesium: SS-EN ISO 11885-1; sulfate: SS-EN ISO 10304-1:2009; turbidity: SS-EN ISO 7027 utg 3, iron: SS-EN ISO 11885-1, manganese: SS-EN ISO 11885-1). Total chlorine was measured with a portable colorimeter (Hach).

### Generation of amplicons

DNA was extracted from cotton swabs or 200 μL resuspended biofilm material using the Fast DNA Spin Kit for Soil (MP Biomedicals) and a bead beater. DNA from two cotton swabs was extracted for each sample, pooled together, and diluted 10-fold before PCR amplification. DNA was also extracted from empty cotton swabs and MilliQ water as negative controls for PCR. PCR amplification of a specific bacterial 16S rRNA region was performed using the primers 27F (5′ GS FLX Titanium adapter A - TCAG - MID - AGAGTTTGATCCTGGCTCAG 3′) and 534R (5′ GS FLX Titanium adapter B - TCAG - MID - ATTACCGCGGCTGCTGGC 3′) (16) with adapter A representing the forward 454 sequencing primer, adapter B representing the 454 reverse primer, and a 10-bp long sample-specific barcode (MID). Barcodes recommended by Roche were used with the following sequences: ACGAGTGCGT (MID1 for P1), ACGCTCGACA (MID2 for P2), AGACGCACTC (MID3 for WM 4), AGCACTGTAG (MID4 for WM 3), ATCAGACACG (MID5 for WM 1), and ATATCGCGAG (MID6 for WM 2). PCR reactions were carried out in a C1000 thermal cycler (Bio-Rad) and contained: 1 × PicoMaxx Reaction buffer, 2 mM MgCl_2_ , 0.2 mM of each dNTP, 0.8 μM of each forward and reverse primer, 1.5 U PicoMaxx polymerase, and 5 μL of template DNA (diluted 10-fold) in a total volume of 30 μL. The cycling parameters were: 5 min at 95°C, followed by 30 cycles of 95°C for 1 min, 55°C for 1 min, 72°C for 1 min, and a final 72°C held for 5 min. Two to three PCR reactions were carried out for each biofilm sample, and pooled together for amplicon purification. The FAST DNA Spin Kit for Soil has been proposed as the method of choice for DWDS DNA extraction, generating representative community information and reproducibility, while PicoMaxx polymerase efficiently amplifies low amounts of DNA in the presence of PCR inhibitors (15, 18). A high concentration of primers was used to outcompete partially extended primers and reduce the formation of chimeras (36).

### Amplicon library preparation

Pooled amplicons were purified using the E.Z.N.A^®^ Cycle Pure Kit (OMEGA, Bio-tek) and Cycle-Pure Spin Protocol according to the manufacturer’s instructions. Sequencing was conducted at the Lund University Sequencing Facility, Sweden. Pooled amplicons were reduced for short fragments by using Agencourt AMPure XP (Beckman Coulter) and inspected using a DNA 1000 kit on a 2100 Bioanalyzer (Agilent). Amplicons were quantified using the Quant-iT dsDNA assay kit (Invitrogen) and Quantifluor fluorometer (Promega), and pools were diluted to obtain a total of 1×10^7^ copies μL^−1^. Titration and library production (aiming at 10–15% enrichment) were performed using emulsion PCR and the Lib-A kit (Roche). DNA-positive beads were enriched, counted on an Innovatis CASY particle counter (Roche), processed using the XLR70 sequencing kit (Roche), and loaded onto a picotiter plate for pyrosequencing on a 454 Life Sciences Genome Sequencer FLX machine (Roche). DNA sequences were archived at NCBI SRA under the accession number SRP039011.

### Data analysis

Amplicons were sequenced from both directions, with reads from forward (V1–V2 region) and reverse (V3 region) directions being treated separately during data analysis following the cleaning step (Supplemental Fig. S1). Reads were sorted by a barcode using sfffile (SFF Tools, Roche). The barcode and TCAG-tags were also removed using sfffile. Reads were quality filtered using PrinSeq lite (v 0.19.3) (34) and custom Perl scripts and discarded if they were shorter than 220 bp, longer than 650 bp, contained ambiguous base pairs, or had a mean quality score below 25. Reads were trimmed after 350 bp or if the mean quality score within a 50 bp sliding window with a step size of 1 was less than 35. Reads with a perfect match to the primer in the 5′ region (27F or 534R) were kept and the sequence information corresponding to the primer region was removed. Reads from the reverse direction were converted to their reverse complement using custom Perl scripts. Chimera checking was done using Uchime (7) in the *de novo* mode after de-replication as implemented in Usearch (v 5.2.32) (6). Reads detected as chimeras were removed from the dataset. In order to avoid bias, sequences were randomly subsampled without replacement to the smallest sample size using the Perl script daisychopper.pl (http://www.genomics.ceh.ac.uk/GeneSwytch/Tools.html v0.6) (11). The subsampled sequences were classified using the command line version of the RDP classifier (v. 2.5) (43) and clustered using CROP (v133) with the option -s corresponding to a 97% sequence identity (14). The parameters were 3176 for -b and 480 for -z for both forward and reverse reads. CROP was run on the Lunarc supercomputer at Lund University. Only clusters with at least 20 sequences in one of the six samples were kept to construct a phylogenetic tree. Metaxa (v 1.1) was applied to detect sequences of chloroplasts, mitochondria, archaea, and eukaryotes (2). Sequences were aligned using Greengenes (4) (greengenes.lbl.gov), and a phylogenetic tree was constructed using RAxML (38) and displayed in iTOL (24). OTUs detected by Metaxa as being chloroplasts or having an uncertain origin, or OTUs that did not align to the Greengenes reference dataset were removed before construction of the phylogenetic tree (see Supplemental Fig. S2).

Venn diagrams were constructed using information from the OTU tables and plotted with the Venn Diagram Plotter (http://omics.pnl.gov/software/VennDiagramPlotter.php). A heatmap of the 50 most abundant OTUs was constructed using the pheatmap package in R (http://cran.r-project.org/web/packages/pheatmap/pheatmap.pdf).

## Results

### 16S rRNA gene amplicon NGS of DWDS biofilms

Six biofilm samples from a single DWDS were selected for a detailed community analysis using NGS of the 16S rRNA gene. The aim was to permit comparisons of both dominant and rare members of the communities to determine whether the community changed with location as well as the number of sequences required to resolve these differences between samples.

**Fig. 1:**
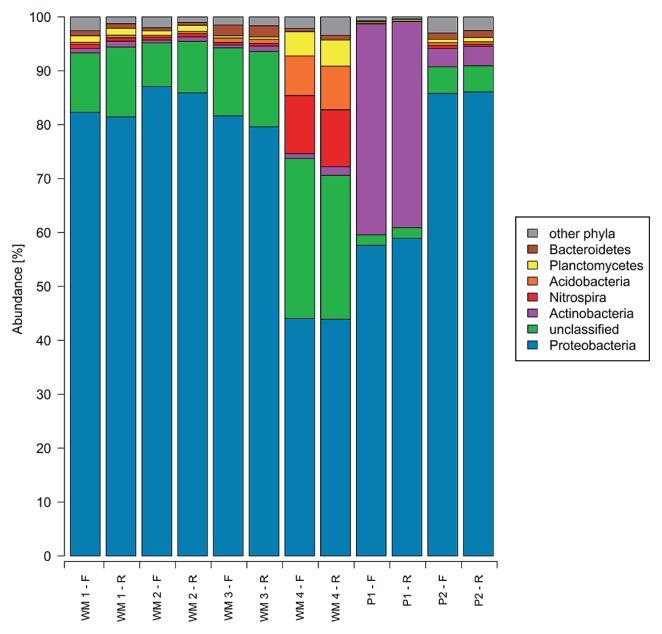
Relative abundance of bacterial phyla from water meters and pipes. F represents the forward sequencing direction covering the V1–V2 region of the bacterial 16S rRNA gene and R represents the reverse sequencing direction covering the V3 region of the bacterial 16S rRNA gene.

**Fig. 2:**
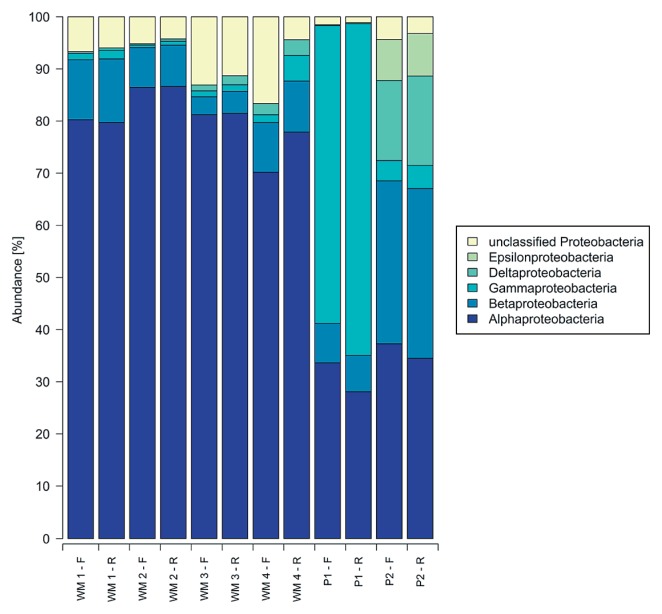
Relative abundance of *Proteobacteria* from water meters and pipes. 100% corresponds to all sequences classified as *Proteobacteria*. F represents the forward sequencing direction covering the V1–V2 region of the bacterial 16S rRNA gene and R represents the reverse sequencing direction covering the V3 region of the bacterial 16S rRNA gene.

Sequences obtained from the forward reads encompassed the V1–V2 region of the 16S rRNA gene while reverse reads corresponded to the V3 region. A total of 674,116 reads describing the six bacterial biofilm communities were initially obtained; 174,817 of these reads did not meet the quality requirements and were removed, and random subsampling selected 52,932 sequences (26,466 for each read direction) to represent each biofilm community (Table 3). Sequences used for analyses ranged in length from 200–332 bp, with an average length of 312 bp after trimming and cleaning. Sequences describing either the V1–V2 region or V3 region were classified using the RDP classifier at a confidence level of 80%. Classification of the V1–V2 and V3 regions showed highly similar trends with respect to the community composition for each biofilm sample at the phylum (Fig. 1) and class (Fig. 2) level.

**Fig. 3:**
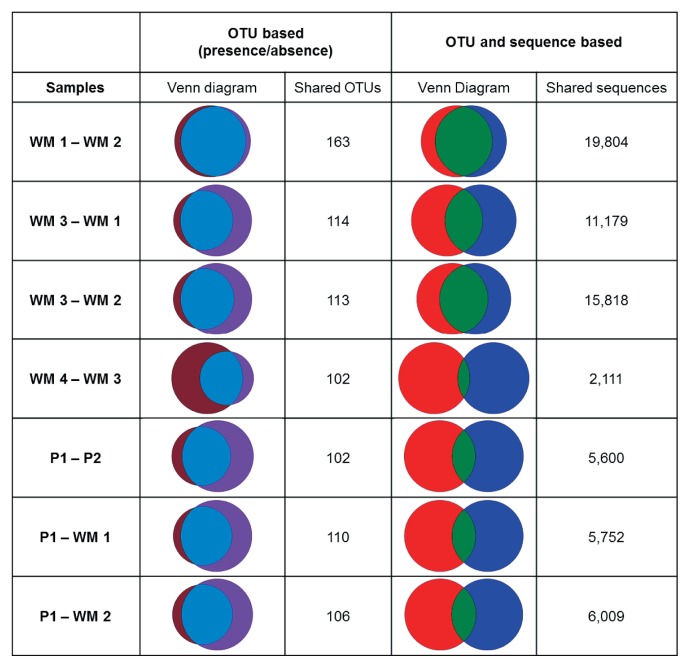
Comparison of OTUs and sequences for selected sample combinations. Venn diagrams are shown comparing the presence and absence of OTUs and shared sequences for the V1–V2 region for selected combinations of samples. The left circle represents the first of the two samples listed in the first column. Only OTUs with at least 20 sequences in one of the six samples were considered in the presence/absence comparison (left panel). The number of OTUs in each sample can be found in Table 3. All OTUs and sequences were considered in the sequence-based comparison (right panel). Each sample contained 26,466 sequences. The number of shared sequences between samples was determined for each OTU and then summed to give the total number of all shared sequences for the samples being compared.

**Fig. 4:**
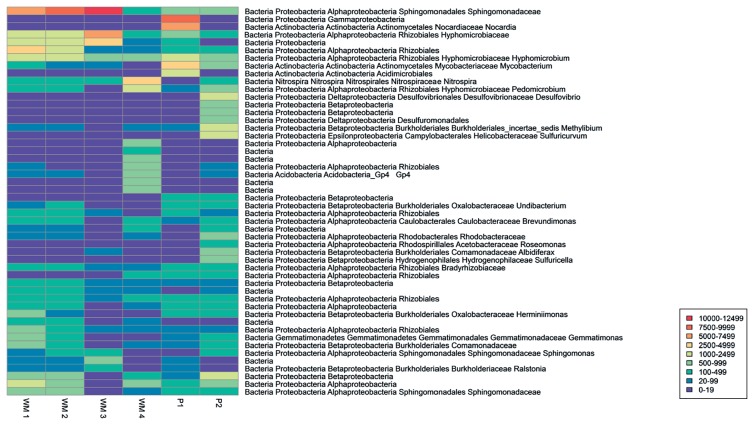
Heatmap of the 50 most abundant OTUs for the V1–V2 region. High similarity was observed in the number of sequences for the selected OTUs for WM1 and WM2, while other samples were distinguished by the presence or absence of specific OTUs. The legend shows the number of sequences corresponding to the different colors of the heatmap.

Sequences were clustered into OTUs (phylotypes), applying a threshold corresponding to 97% identity. OTUs relevant for comparisons of the distinct biofilm bacterial communities were restricted to those containing 20 or more sequences from any one of the six biofilm samples in order to avoid including sequences containing errors introduced during sequencing or PCR amplification (17). The removal of rare sequences reduced the number of OTUs from 2,383 to 308. The number of OTUs representing each of the six biofilm communities ranged from 126 to 227 (Table 3, V1–V2 region) with 57 of these 308 OTUs being present in all the biofilm communities examined. Since comparisons of information obtained from the V1–V2 and V3 regions by both RDP classification (Fig. 1 and Fig. 2) and OTU frequencies (Fig. 3, 4, Table 4 and 5) gave similar results, the V1–V2 region was chosen to facilitate comparisons between the different biofilm communities.

### Assessment of reproducibility using biofilm communities from parallel water meters

Bacterial biofilm communities in water meters (WM) installed in parallel within the DWDS were physically present on distinct surfaces of two water meters (WM 1 and WM 2), and had developed for four years within an apartment building that had experienced identical temperatures, seasons, water flow, and source water (Supplemental Fig. S3). The analysis of these two communities was used to examine the reproducibility of the established high resolution sequencing work-flow for determining community compositions, OTU frequencies, and phylotypes.

WM 1 and WM 2 were dominated at the phylum level by *Proteobacteria* (82% for WM 1; 87% for WM 2), and unclassified Bacteria (11% for WM 1; 8% for WM 2, Fig. 1). A total of 185 OTUs were identified in WM 1 and 177 OTUs in WM 2, with the two communities sharing 163 OTUs and 75% of the sequences (Fig. 3). A heatmap illustrating the 50 most abundant OTUs showed highly similar profiles of OTU frequencies for both water meters (Fig. 4).

The phylotype-based analysis showed that the most abundant OTU was classified to the family level as *Sphingomonadaceae* in both WM 1 (22%) and WM 2 (36%). Other abundant OTUs were *Hyphomicrobium* (WM 1: 5% and WM 2: 6%) and unclassified *Proteobacteria* (WM 1: 6% and WM 2: 5%).

To determine whether the bacterial biofilm community changed within a single DWDS or if the water meter biofilms were similar throughout the same DWDS, biofilms were sampled from a third water meter (WM 3) connected to the same distribution system a few kilometers away from the WM 1 and 2 sampling site.

WM 3 was also dominated by *Proteobacteria* and gave a similar overall picture at the phylum and class levels as WM 1 and WM 2 (Fig. 1 and Fig. 2). Sequences from WM 3 were classified into the 20 most abundant OTUs in largely the same proportions as those classified for WM 1 and WM 2 (Table 4). These included OTUs of *Sphingomonadaceae* (40%), *Hyphomicrobiaceae* (20%), and unclassified *Proteobacteria* (13%). The diversity of WM 3 was represented by slightly fewer OTUs (a total of 129) than WM 1 and WM 2 (185 and 177, respectively) (Table 3). WM 3 shared 114 OTUs and 42% of the sequences with WM 1, and 113 OTUs and 60% of the sequences with WM 2 (Fig. 3).

### A bacterial biofilm from an area with unacceptable water quality

A biofilm from a fourth water meter within the same DWDS was sampled from a location in which problems with water quality had been reported by the consumer in order to determine whether changes within the bacterial biofilm community associated with changes in water quality could be resolved.

Differences were observed at the phylum level between WM 4 and WM 1–3. *Proteobacteria* were less abundant than in the other water meters (44% in WM 4 and 82–87% in WM 1–3). However, the composition of classes within the *Proteobacteria* remained the same across all WMs with *Alphaproteobacteria* as the most abundant class (Fig. 2). *Planctomycetes* (4%), *Acidobacteria* (7%), and *Nitrospira* (11%) were present in WM 4 and virtually absent in any of the other samples. The amount of unclassified bacteria in WM 4 was the highest at 30%, while these amounts were only 2–13% in the other five samples. Within the 20 most abundant OTUs, only 1% of the WM 4 sequences belonged to the OTU classified as family *Sphingomonadaceae.*

Certain OTUs contained a large number of sequences from WM 4, but were not represented in other WMs, including sequences classified as genus *Nitrospira* (11%) and genus *Pedomicrobium* (7%) for WM 4.

WM 4 contained 227 OTUs, the highest number of OTUs found in any of the six biofilm samples. WM 4 and WM 3 shared 102 OTUs, similar to the amount of shared OTUs observed between other samples (Fig. 3); however, only 8% of the sequences were shared. Differences in the abundance of the sequences with the OTUs obtained from WM 4 differentiated this bacterial biofilm community from those of WM 1–3: the heatmap profile of OTU frequency (Fig. 4) showed that the distribution of sequences across OTUs was distinct for WM 4.

### Bacterial community composition in DWDS pipes

A biofilm was sampled from a pipe (P1) to compare the pipe bacterial biofilm community adjacent to the biofilm communities established on water meters. A second pipe (P2) was selected as a biofilm community from the same DWDS, but with a number of distinct characteristics such as the age of the biofilm (Table 1).

An analysis at the phylum level (Fig. 1) showed that P1 was dominated by *Proteobacteria* (58%) and *Actinobacteria* (39%), with other phyla accounted for less than 3% of the sequences.

The classification identified specific OTUs containing large numbers of sequences that were mainly found in P1. These included class *Gammaproteobacteria* (33%); order *Acidomicrobiales* (7%); and within order *Actinomycetales*, *Mycobacterium* (13%) and *Nocardia* (19%). P1 contained fewer sequences belonging to the family *Sphingomonadaceae* (2%) than those describing WM 1–3.

P1 contained 126 OTUs, the fewest OTUs found in any of the samples examined, sharing 110 OTUs with WM 1 and 106 OTUs with WM 2 (Fig. 3). As observed with WM 4, despite the number of shared OTUs between samples, P1 shared fewer sequences with WM 1 (21%) and WM 2 (23%).

At the phylum level, sequences obtained from P2 consisted of 86% *Proteobacteria* and 5% unclassified Bacteria with other phyla accounting for less than 3%, as observed for P1. Within *Proteobacteria*, both pipes consisted of sequences more widely distributed across the different classes, and were not predominated by *Alphaproteobacteria* as observed for WM 1–3 and WM 4 (Fig. 2). A small overlap in the community structure was observed between P1 and P2. OTU frequencies gave a heatmap profile of P2 that was distinct from all other samples, with the highest OTU frequencies appearing in OTUs that were not predominant in any other sample examined; for example *Desulfovibrio* (9%), and *Sulfuricurvum* (6%) (Fig. 4), and, although a number of the OTUs themselves were shared, only 21% of the sequences were shared between P1 and P2 (Fig. 3).

## Discussion

The bacterial communities present in six samples from a single DWDS system in Sweden were analyzed using amplicon NGS of the V1–V2 and V3 regions of the 16S rRNA gene. Sampling sites were chosen to test the resolution and limits of the experimental and analytical protocols, and to determine whether the described work-flow was capable of resolving community changes associated with small variations in a DWDS ecosystem.

Although the most relevant analysis of drinking water biofilms is an examination of those within established drinking water delivery networks, one difficulty associated with working in these systems is obtaining biological replicates to validate the experimental approach. In this study, bacterial biofilm communities of parallel installed water meters (WM 1, WM 2) allowed an approach to be developed and tested for a high resolution community analysis through NGS that included controls for both the experimental approach, by comparing results between WM 1 and WM 2, as well as the influence of changes in the DWDS on the community, by comparing additional drinking water biofilms.

Biofilms from virtually identical locations and sampling regimes associated with WM 1 and WM 2 showed highly similar communities when described by any of the NGS analyses in this study. This degree of similarity between two descriptions of communities has not been reported in previous studies that used NGS to examine the bacterial communities of drinking water biofilms, and may reflect the difficulty in obtaining true biological replicates in working DWDSs. Even small variations in the ecology of the drinking water biofilm can affect the community composition. Hong *et al.* (16) observed numerous differences in communities associated with two water meters that had experienced stable turbidity, pH, and chlorine levels, but differed in the origin of the water meter (two separate households) and the time of year they were sampled (October and December). Douterelo *et al.* (5) examined biofilm communities within a model DWDS, including NGS of material assumed to be biological replicates; however, even samples retrieved from this highly homogenous model system produced biofilm communities that showed a high variability in biological diversities across three biological replicates. The diversity was suggested to have been linked to the short time (28 d) over which the biofilms were grown, and Martiny *et al.* (29) showed that biofilms in a model drinking water system followed a successional formation that only culminated in a stable population after three years. Thus, the high similarity between WM 1 and WM 2 is a reflection of the nearly identical physical and temporal parameters that nurtured the ecology of these two biofilms.

This result strongly suggests that the differences observed in the other WM communities arose from ecological changes and not from experimental or analytical artefacts and, in addition to physical parameters defining the ecosystem, the time over which this biofilm developed also contributed to the consistency of the observed community composition. The results obtained for WM 1 and WM 2 suggest that differences in the WM 3 composition were related to changes in the environment of WM3; small differences in the community, OTU, and phylotype comparisons may be due to changes in the geographical location of WM 3 and/or water consumption associated with this biofilm community relative to WM 1 and WM 2. The biofilms in WM 1 and WM 2 were obtained from a building with high water consumption (436 and 527 m^3^ over six months, respectively), whereas that in WM 3 was obtained from a water meter within a family house that had a lower water consumption of 49 m^3^ over six months. The communities in WM 4, and P1 and P2 pipe biofilms, may have diverged from those of WM 1–3 to the degree that the surrounding environment of the biofilm differed. Since the environment of WM 4 was the most similar to that of WM 3 (Table 1), differences in this community may be explained by its unique location within the DWDS, and/or the distinct water quality profile associated with this sample. The composition of biofilms was different in pipe biofilm communities and those in water meters, while similar OTUs were present in both types of samples, but only a low percentage of sequences were shared (Fig. 3). The hydrological characteristics (*i.e.* pipe diameter), geographical location (including temperature), sampling season, microbial corrosion, and age of the biofilm differed between P1 and P2, which made it difficult to define any relationship between community composition and any DWDS or sampling parameters.

In addition, sampling of pipe biofilms within a working DWDS is often determined by the water company’s activities, which further limits study design. The location of the water meter biofilm at the boundary between the drinking water provider and consumer often represents a change in responsibility for water quality and, together with the results presented here, support and strengthen the application of sampling water meters for biofilm communities in contact with drinking water. In contrast, water meters contain structures and surfaces for the establishment of biofilms that are the same at each sampling location; therefore, the possibility exists for biological replicates within a DWDS if parallel water meters have been installed in some buildings, and installation and removal is simple and monitored so the length of time for the biofilm to have become established is known. Hong *et al.* (16) also proposed that biofilms obtained from water meters were suitable for studying the microbial ecology of DWDS due to the ease of sampling. With uniform physical structures and the possibility for more standardized sampling, comparisons of WM communities can more directly be related to variables such as water quality and/or water usage.

Large numbers of sequences that clustered into OTUs described as *Sphingomonadaceae* were found in the water meter samples WM 1–3, with a reduced number of sequences being detected for this group in WM 4. *Sphingomonadaceae* have previously been detected in drinking water systems (16, 44) and have been related to drinking water quality because they may be responsible for initial biofilm formation (3) and are very resistant to chlorine (39). These phenotypes may promote bacterial growth in the distribution system and, thus, influence drinking water quality. The WM 4 community had a reduced number of sequences that were classified as *Sphingomonadaceae*, and a community composition distinct from the other water meters in this study. This was also observed at phylum level, with *Proteobacteria*, the most abundant phylum in the drinking water biofilms analyzed in this study and others (16, 25, 33, 44), being markedly reduced in WM 4. A decrease in bacterial biofilm diversity has been associated with a loss of multifunctionality (31) and the larger number of OTUs observed at WM 4 may reflect a more diverse substrate or more active biofilm using the wider spectrum of nutrients present in less-than-ideal drinking water.

Since water consumption was similar between WM 3 and 4 (49 m^3^ in six months for WM 3 and 53 m^3^ in 6 months for WM 4), other factors may account for the differences observed between these biofilm communities.

Sequences related to *Nitrospira* were more abundant in WM 4 than in WM 1–3. Ling and Liu (26) observed a community shift to *Nitrospirae* in unchloraminated biofilms, suggesting the sensitivity of *Nitrospirae* to the disinfection treatment, and *Nitrospira* was previously detected in a model DWDS using unchlorinated groundwater (29). Even though chlorination has been used for disinfection in the DWDS examined in this study, water samples characterizing flowing water moving past the WM 4 biofilm had a lower total chlorine concentration (0.05 mg L^−1^ Cl_2_ ) than those for the other WM communities (WM 1, WM 2: 0.13 mg L^−1^ Cl_2_ ; WM 3: 0.14 mg L^−1^ Cl_2_ ). Lower chlorine levels may be related to the report of red water for WM 4, as an increase in biofilm activity (due to reduced exposure to a disinfectant) has been associated with an increase in the deposition of iron and manganese into biofilms (12). Cell death within the biofilms or disruption due to hydraulic changes may release deposited iron and manganese, resulting in red water and the elevated levels of iron and manganese previously observed during discolored water events (35). Li *et al.* (25) reported changes in the bacterial community associated with the occurrence of red water and observed a higher percentage of the iron-oxidizing bacteria *Gallionella* together with the extensive precipitation of iron oxides in water samples. Although *Gallionella* was not detected in the present study, *Pedomicrobium* was identified in WM 4, the only community that was exposed to flowing water containing detectable iron and manganese concentrations (1.5 mg L^−1^ Fe and 0.04 mg L^−1^ Mn). The presence of these metals within the water may support the growth of *Pedomicrobium*, which deposits oxidized metals on its cell surface, resulting in the accumulation of metal oxides in biofilms (23, 32).

## Conclusion

This study has established a work-flow that has the ability to resolve biofilm communities in sufficient detail to permit their composition to be related to the ecology of real DWDS biofilms; therefore, a definitive relationship between bacterial community compositions may be established by using this approach with a large number of biofilm samples representing diverse properties and qualities of both drinking water and its distribution systems. The high reproducibility observed between the two parallel installed water meters and other water meters from the same city suggests that water meters are an appropriate sampling site if the aim is to compare different locations within a DWDS. This study demonstrated that differences between drinking water biofilms were observable at the phylum level, which indicated that sequencing of the V1–V2 region of the 16S rRNA gene may provide sufficient information regarding bacterial community compositions for high throughput analyses and comparisons. Lundin *et al.* (28) suggested that 5,000 sequences allowed trends in alpha diversity to be estimated; hence, using fewer sequences from a single 16S region and biofilm from easily accessible water meters will facilitate the analysis of many bacterial biofilm communities associated with differing water qualities. The results of the present study support this experimental and analytical approach as a strategy for compiling an accurate and complete knowledge of the ecology of DWDS biofilms and their role in drinking water delivery.

## Supplementary Information



## Figures and Tables

**Table 1:** Samples analyzed in this study. Samples were taken in the city of Landskrona, Sweden, in which surface water is used to produce drinking water. All water meters had a rated flow of Qn 2.5 m^3^ h^−1^. A photo showing the parallel water meters (WM1 and WM 2) is included as Supplemental Fig. S3.

Sample	Type	Diameter	Age	Material	DNA extraction	PCR reactions	Sampling date	Description
WM 1 WM 2	water meter	n/a	2007	brass and plastic	Two cotton swabs	2	March 2011	Water meters installed in parallel in an apartment building
WM 3	water meter	n/a	2001	brass and plastic	Two cotton swabs	3	March 2011	Water meter from a family house
WM 4	water meter	n/a	2004	brass and plastic	Two cotton swabs	2	March 2011	Water meter from a family house, in which problems with red water have been reported
P1	pipe	50 cm	1966	cast iron	Two extractions from 200 μL of resuspended biofilm material	3	April 2011	Pipe situated in the same street as WM 1 and WM 2
P2	pipe	15 cm	1908	cast iron	Two extractions from 200 μL of resuspended biofilm material	3	June 2011	Pipe situated in the Landskrona DWDS

**Table 2:** Water Quality Parameters. Water was obtained from households in which different water meter sampling was conducted and submitted for a routine analysis. Representative water quality data for the city of Landskrona (Sweden) are included for comparisons. The water of WM 4 had a brown color during sampling.

	Landskrona	WM 1 + 2	WM 3	WM 4
pH	8.1	8.1	8	8.1
Conductivity [mS m^−1^]	40.2	40.2	38.4	39.7
Hardness [°dH]	9.5	11	8.7	8.9
Calcium Ca [mg L^−1^]	63	70	58	59
Magnesium Mg [mg L^−1^]	3.1	3.4	2.9	2.9
Sulfate SO_4_^2−^ [mg L^−1^]	—	83	80	85
Turbidity [FNU]	0.21	0.24	<0.1	12
Iron Fe [mg L^−1^]	<0.05	<0.05	<0.05	1.5
Manganese Mn [mg L^−1^]	<0.02	<0.02	<0.02	0.04
Total chlorine [mg L^−1^ Cl_2_ ]	—	0.13	0.14	0.05

**Table 3:** Overview of NGS Results. F represents the forward sequencing direction covering the V1–V2 region of the bacterial 16S rRNA gene and R represents the reverse sequencing direction covering the V3 region.

	chimeras	cleaned sequences	subsampled	OTUs	singletons	doubletons	OTUs^a^
WM 1 - F	218	35,662	26,466	824	289	159	185
WM 2 - F	212	30,346	26,466	669	260	108	177
WM 3 - F	160	45,433	26,466	923	376	171	129
WM 4 - F	247	26,466	26,466	818	218	123	227
P1 - F	189	30,701	26,466	282	93	45	126
P2 - F	495	26,695	26,466	622	198	106	184
WM 1 - R	638	51,553	26,466	622	239	105	154
WM 2 - R	510	42,893	26,466	537	209	106	151
WM 3 - R	271	66,700	26,466	709	275	128	133
WM 4 - R	590	42,174	26,466	543	162	82	165
P1 - R	205	53,023	26,466	216	82	30	105
P2 - R	1,611	42,307	26,466	458	152	66	144

a OTUs in which at least one of the six samples contained 20 sequences.

**Table 4:** Classification of the 20 most abundant OTUs for the V1–V2 region. OTUs are from the bacterial 16S rRNA gene found in the six drinking water biofilm samples from water meters and pipes. Sequences for the OTUs are found in Supplemental Table S1.

Phylum	Class	Order	Family	Genus	WM 1 - F	WM 2 - F	WM 3 - F	WM 4 - F	P1 - F	P2 - F
*Actinobacteria*	*Actinobacteria*	*Acidimicrobiales*			0	0	0	0	1,875	0
*Actinobacteria*	*Actinobacteria*	*Actinomycetales*	*Mycobacteriaceae*	*Mycobacterium*	118	69	81	4	3,369	859
*Actinobacteria*	*Actinobacteria*	*Actinomycetales*	*Nocardiaceae*	*Nocardia*	2	3	2	3	5,050	2
*Gemmatimonadetes*	*Gemmatimonadetes*	*Gemmatimonadales*	*Gemmatimonadaceae*	*Gemmatimonas*	523	318	6	7	87	141
*Nitrospira*	*Nitrospira*	*Nitrospirales*	*Nitrospiraceae*	*Nitrospira*	196	146	117	2,852	2	137
*Proteobacteria*	*Alphaproteobacteria*	*Rhizobiales*	*Hyphomicrobiaceae*	*Hyphomicrobium*	1,401	1,715	580	751	1,701	664
*Proteobacteria*	*Alphaproteobacteria*	*Rhizobiales*	*Hyphomicrobiaceae*	*Pedomicrobium*	488	363	8	1,949	78	553
*Proteobacteria*	*Alphaproteobacteria*	*Rhizobiales*	*Hyphomicrobiaceae*		1,144	2,332	5,171	102	543	380
*Proteobacteria*	*Alphaproteobacteria*	*Rhizobiales*			3,723	2,263	59	44	321	423
*Proteobacteria*	*Alphaproteobacteria*	*Rhizobiales*			378	336	55	109	146	205
*Proteobacteria*	*Alphaproteobacteria*	*Rhizobiales*			570	312	89	76	99	71
*Proteobacteria*	*Alphaproteobacteria*	*Sphingomonadales*	*Sphingomonadaceae*		5,952	9,578	10,689	149	581	604
*Proteobacteria*	*Alphaproteobacteria*	*Sphingomonadales*	*Sphingomonadaceae*		935	617	17	48	160	369
*Proteobacteria*	*Alphaproteobacteria*				1,084	807	12	715	265	623
*Proteobacteria*	*Betaproteobacteria*				753	579	10	334	98	1,466
*Proteobacteria*	*Betaproteobacteria*	*Burkholderiales*	*Burkholderiales_incertae_sedis*	*Methylibium*	85	27	7	33	24	1,434
*Proteobacteria*	*Deltaproteobacteria*	*Desulfovibrionales*	*Desulfovibrionaceae*	*Desulfovibrio*	1	1	0	0	1	2,470
*Proteobacteria*	*Epsilonproteobacteria*	*Campylobacterales*	*Helicobacteraceae*	*Sulfuricurvum*	1	0	0	0	0	1,476
*Proteobacteria*	*Gammaproteobacteria*				0	0	2	0	8,621	0
*Proteobacteria*					1,542	1,258	3,490	56	106	13

				Percentage of total sequences	71%	78%	77%	27%	87%	45%
					

**Table 5:** Classification of the 20 most abundant OTUs for the V3 region. OTUs are from the bacterial 16S rRNA gene found in the six drinking water biofilm samples from water meters and pipes. Sequences for the OTUs are found in Supplemental Table S1.

Phylum	Class	Order	Family	Genus	WM 1 - R	WM 2 - R	WM 3 - R	WM 4 - R	P1 - R	P2 - R
*Actinobacteria*	*Actinobacteria*	*Acidimicrobiales*			0	3	0	2	1,630	1
*Actinobacteria*	*Actinobacteria*	*Actinomycetales*			146	106	103	101	8,445	919
*Nitrospira*	*Nitrospira*	*Nitrospirales*	*Nitrospiraceae*	*Nitrospira*	168	157	119	2,800	5	94
*Proteobacteria*	*Alphaproteobacteria*	*Caulobacterales*	*Caulobacteraceae*		998	667	6	677	193	523
*Proteobacteria*	*Alphaproteobacteria*	*Rhizobiales*	*Hyphomicrobiaceae*		7,398	6,937	5,832	4,007	2,645	2,530
*Proteobacteria*	*Alphaproteobacteria*	*Rhizobiales*			365	251	10	145	247	432
*Proteobacteria*	*Alphaproteobacteria*	*Sphingomonadales*	*Sphingomonadaceae*		6,913	10,667	10,808	254	614	1,166
*Proteobacteria*	*Alphaproteobacteria*				257	217	75	1,175	55	400
*Proteobacteria*	*Alphaproteobacteria*				253	206	53	1,204	104	235
*Proteobacteria*	*Betaproteobacteria*	*Burkholderiales*	*Comamonadaceae*		619	331	53	46	53	1,015
*Proteobacteria*	*Betaproteobacteria*	*Burkholderiales*	*Oxalobacteraceae*	*Herminiimonas*	562	420	21	38	375	409
*Proteobacteria*	*Betaproteobacteria*	*Burkholderiales*			131	30	16	62	49	1,800
*Proteobacteria*	*Betaproteobacteria*	*Rhodocyclales*	*Rhodocyclaceae*		259	170	52	245	102	629
*Proteobacteria*	*Betaproteobacteria*				840	639	10	369	92	1,599
*Proteobacteria*	*Deltaproteobacteria*	*Desulfovibrionales*	*Desulfovibrionaceae*	*Desulfovibrio*	2	0	0	0	0	2,779
*Proteobacteria*	*Epsilonproteobacteria*	*Campylobacterales*	*Helicobacteraceae*	*Sulfuricurvum*	4	2	0	0	0	1,854
*Proteobacteria*	*Gammaproteobacteria*				0	2	12	0	9,805	0
*Proteobacteria*					1,588	1,225	3,596	38	65	2
					439	298	21	905	87	141
					0	0	0	1,820	0	0

				Percentage of total sequences	79%	84%	79%	52%	93%	62%
					

## References

[1] Amann RI, Ludwig W, Schleifer KH (1995). Phylogenetic identification and *in situ* detection of individual microbial cells without cultivation. Microbiol Rev.

[2] Bengtsson J, Eriksson KM, Hartmann M, Wang Z, Shenoy BD, Grelet GA, Abarenkov K, Petri A, Rosenblad MA, Nilsson RH (2011). Metaxa: a software tool for automated detection and discrimination among ribosomal small subunit (12S/16S/18S) sequences of archaea, bacteria, eukaryotes, mitochondria, and chloroplasts in metagenomes and environmental sequencing datasets. Antonie Van Leeuwenhoek.

[3] Bereschenko LA, Stams AJ, Euverink GJ, van Loosdrecht MCM (2010). Biofilm formation on reverse osmosis membranes is initiated and dominated by *Sphingomonas* spp. Appl Environ Microbiol.

[4] DeSantis TZ, Hugenholtz P, Keller K, Brodie EL, Larsen N, Piceno YM, Phan R, Andersen GL (2006). NAST: a multiple sequence alignment server for comparative analysis of 16S rRNA genes. Nucleic Acids Res.

[5] Douterelo I, Sharpe RL, Boxall JB (2013). Influence of hydraulic regimes on bacterial community structure and composition in an experimental drinking water distribution system. Water Res.

[6] Edgar RC (2010). Search and clustering orders of magnitude faster than BLAST. Bioinformatics.

[7] Edgar RC, Haas BJ, Clemente JC, Quince C, Knight R (2011). UCHIME improves sensitivity and speed of chimera detection. Bioinformatics.

[8] Eichler S, Christen R, Holtje C, Westphal P, Botel J, Brettar I, Mehling A, Hofle MG (2006). Composition and dynamics of bacterial communities of a drinking water supply system as assessed by RNA- and DNA-based 16S rRNA gene fingerprinting. Appl Environ Microbiol.

[9] Flemming HC (2002). Biofouling in water systems-cases, causes and countermeasures. Appl Microbiol Biotechnol.

[10] Flemming HC, Wingender J (2010). The biofilm matrix. Nat Rev Microbiol.

[11] Gilbert JA, Field D, Swift P, Newbold L, Oliver A, Smyth T, Somerfield PJ, Huse S, Joint I (2009). The seasonal structure of microbial communities in the Western English Channel. Environ Microbiol.

[12] Ginige MP, Wylie J, Plumb J (2011). Influence of biofilms on iron and manganese deposition in drinking water distribution systems. Biofouling.

[13] Hall-Stoodley L, Costerton JW, Stoodley P (2004). Bacterial biofilms: from the natural environment to infectious diseases. Nat Rev Microbiol.

[14] Hao X, Jiang R, Chen T (2011). Clustering 16S rRNA for OTU prediction: a method of unsupervised Bayesian clustering. Bioinformatics.

[15] Hedman J, Nordgaard A, Rasmusson B, Ansell R, Rådström P (2009). Improved forensic DNA analysis through the use of alternative DNA polymerases and statistical modeling of DNA profiles. Biotechniques.

[16] Hong PY, Hwang C, Ling F, Andersen GL, LeChevallier MW, Liu WT (2010). Pyrosequencing analysis of bacterial biofilm communities in water meters of a drinking water distribution system. Appl Environ Microbiol.

[17] Huse SM, Welch DM, Morrison HG, Sogin ML (2010). Ironing out the wrinkles in the rare biosphere through improved OTU clustering. Environ Microbiol.

[18] Hwang C, Ling F, Andersen GL, LeChevallier MW, Liu WT (2012). Evaluation of methods for the extraction of DNA from drinking water distribution system biofilms. Microbes Environ.

[19] Hwang C, Ling F, Andersen GL, LeChevallier MW, Liu WT (2012). Microbial community dynamics of an urban drinking water distribution system subjected to phases of chloramination and chlorination treatments. Appl Environ Microbiol.

[20] Kalmbach S, Manz W, Szewzyk U (1997). Isolation of new bacterial species from drinking water biofilms and proof of their *in situ* dominance with highly specific 16S rRNA probes. Appl Environ Microbiol.

[21] Kwon S, Moon E, Kim TS, Hong S, Park H-D (2011). Pyrosequencing demonstrated complex microbial communities in a membrane filtration system for a drinking water treatment plant. Microbes Environ.

[22] Långmark J, Storey MV, Ashbolt NJ, Stenström TA (2005). Accumulation and fate of microorganisms and microspheres in biofilms formed in a pilot-scale water distribution system. Appl Environ Microbiol.

[23] Larsen EI, Sly LI, McEwan AG (1999). Manganese(II) adsorption and oxidation by whole cells and a membrane fraction of *Pedomicrobium* sp. ACM 3067. Arch Microbiol.

[24] Letunic I, Bork P (2007). Interactive Tree Of Life (iTOL): an online tool for phylogenetic tree display and annotation. Bioinformatics.

[25] Li D, Li Z, Yu J, Cao N, Liu R, Yang M (2010). Characterization of bacterial community structure in a drinking water distribution system during an occurrence of red water. Appl Environ Microbiol.

[26] Ling F, Liu WT (2013). Impact of chloramination on the development of laboratory-grown biofilms fed with filter-pretreated groundwater. Microbes Environ.

[27] Liu R, Yu Z, Guo H, Liu M, Zhang H, Yang M (2012). Pyrosequencing analysis of eukaryotic and bacterial communities in faucet biofilms. Sci Total Environ.

[28] Lundin D, Severin I, Logue JB, Ostman O, Andersson AF, Lindstrom ES (2012). Which sequencing depth is sufficient to describe patterns in bacterial alpha- and beta-diversity?. Environ Microbiol Rep.

[29] Martiny AC, Jorgensen TM, Albrechtsen HJ, Arvin E, Molin S (2003). Long-term succession of structure and diversity of a biofilm formed in a model drinking water distribution system. Appl Environ Microbiol.

[30] Nocker A, Richter-Heitmann T, Montijn R, Schuren F, Kort R (2010). Discrimination between live and dead cells in bacterial communities from environmental water samples analyzed by 454 pyrosequencing. Int Microbiol.

[31] Peter H, Ylla I, Gudasz C, Romani AM, Sabater S, Tranvik LJ (2011). Multifunctionality and diversity in bacterial biofilms. Plos One.

[32] Ridge JP, Lin M, Larsen EI, Fegan M, McEwan AG, Sly LI (2007). A multicopper oxidase is essential for manganese oxidation and laccase-like activity in *Pedomicrobium* sp. ACM 3067. Environ Microbiol.

[33] Schmeisser C, Stockigt C, Raasch C (2003). Metagenome survey of biofilms in drinking-water networks. Appl Environ Microbiol.

[34] Schmieder R, Edwards R (2011). Quality control and preprocessing of metagenomic datasets. Bioinformatics.

[35] Seth A, Bachmann R, Boxall J, Saul A, Edyvean R (2004). Characterisation of materials causing discolouration in potable water systems. Water Sci Technol.

[36] Smyth RP, Schlub TE, Grimm A, Venturi V, Chopra A, Mallal S, Davenport MP, Mak J (2010). Reducing chimera formation during PCR amplification to ensure accurate genotyping. Gene.

[37] Staley JT, Konopka A (1985). Measurement of *in situ* activities of nonphotosynthetic microorganisms in aquatic and terrestrial habitats. Annu Rev Microbiol.

[38] Stamatakis A (2006). RAxML-VI-HPC: maximum likelihood-based phylogenetic analyses with thousands of taxa and mixed models. Bioinformatics.

[39] Sun DL, Jiang X, Wu QL, Zhou NY (2013). Intragenomic heterogeneity of 16S rRNA genes causes overestimation of prokaryotic diversity. Appl Environ Microbiol.

[40] Szewzyk U, Szewzyk R, Manz W, Schleifer KH (2000). Microbiological safety of drinking water. Annu Rev Microbiol.

[41] Van Der Kooij D (2000). Biological stability: a multidimensional quality aspect of treated water. Water Air Soil Pollut.

[42] Vaz-Moreira I, Egas C, Nunes OC, Manaia CM (2013). Bacterial diversity from the source to the tap: a comparative study based on 16S rRNA gene-DGGE and culture-dependent methods. FEMS Microbiol Ecol.

[43] Wang Q, Garrity GM, Tiedje JM, Cole JR (2007). Naive Bayesian classifier for rapid assignment of rRNA sequences into the new bacterial taxonomy. Appl Environ Microbiol.

[44] Zhang M, Liu W, Nie X, Li C, Gu J, Zhang C (2012). Molecular analysis of bacterial communities in biofilms of a drinking water clearwell. Microbes Environ.

