# Supplementary material for: Bacterial Community Analysis of Drinking Water Biofilms in Southern Sweden
Source: Microbes Environ. 2015 Feb 21;30(1):99–107. doi: 10.1264/jsme2.ME14123 (PMC4356470; doi:10.1264/jsme2.ME14123)
Supplement: Supplementary file 1 [file 30_99_s1.pdf]

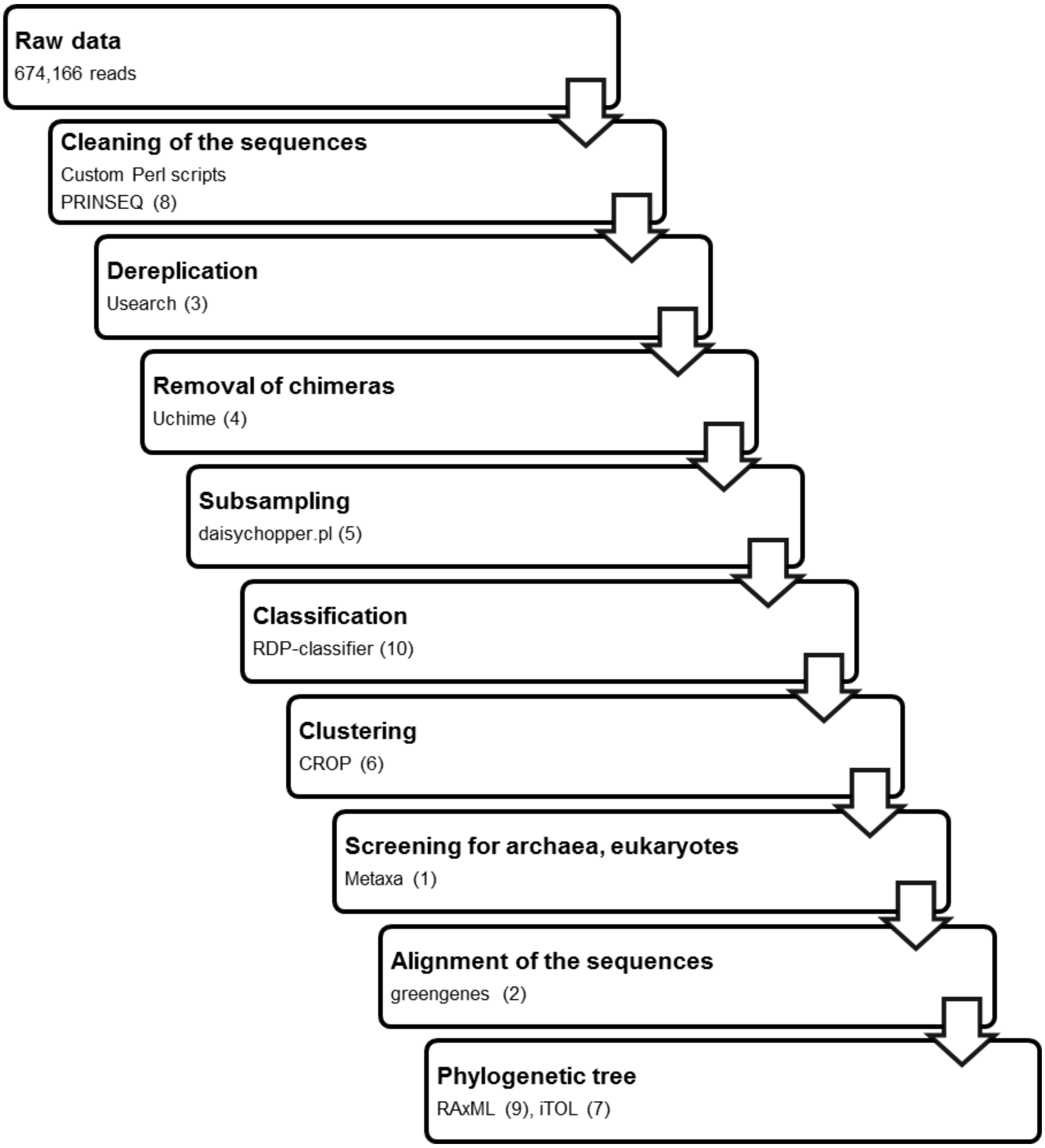

Fig. S1

Description of the bioinformatic work-flow. The number in brackets corresponds to the reference in the reference list describing each program.

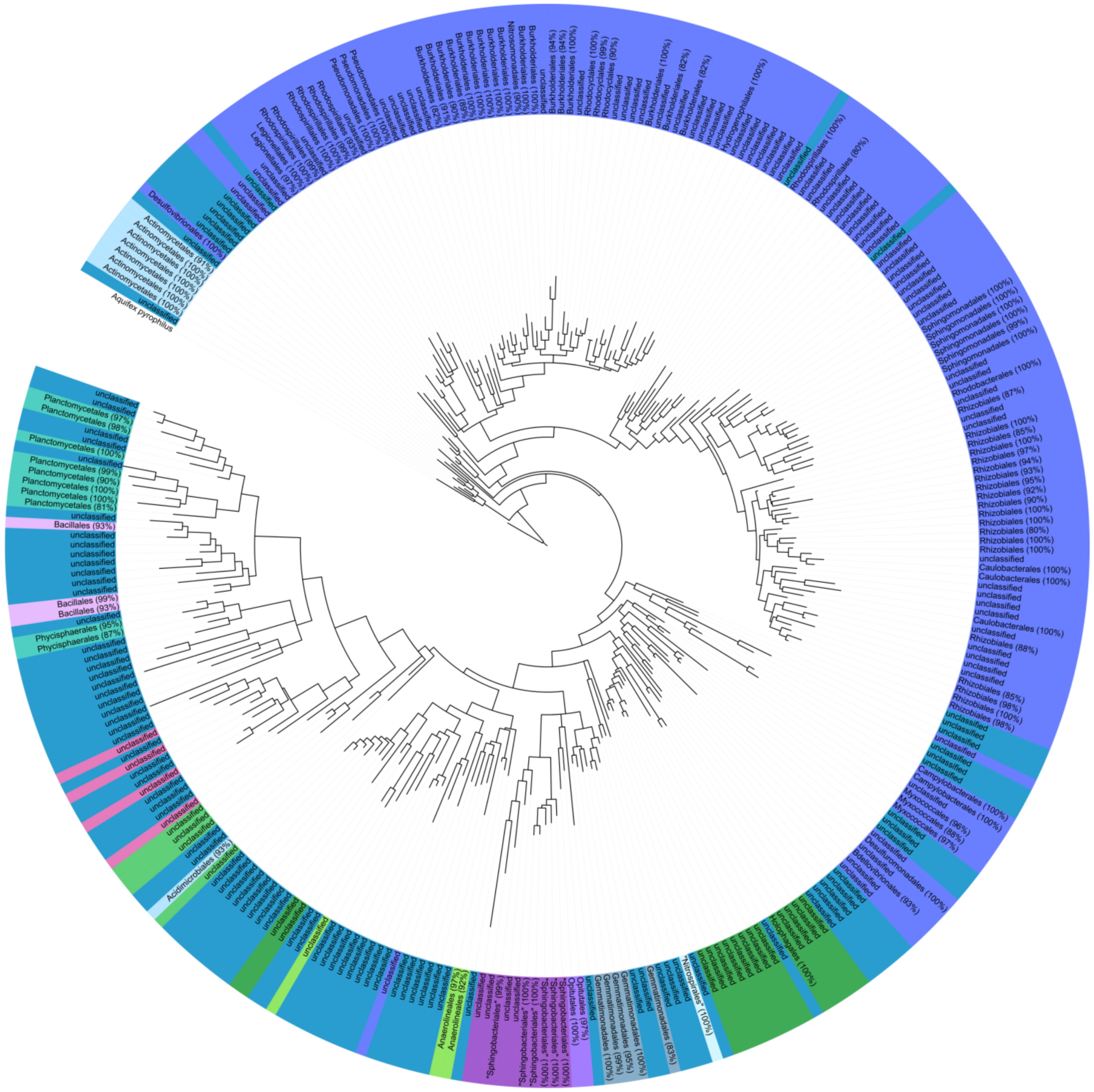

Fig. S2

Phylogenetic tree of all OTUs (299 OTUs, V1-V2 region, 16S rRNA gene) where at least 20 sequences were found in one of the six bacterial drinking water biofilm communities. The colour corresponds to phylum and the label to order level. Classification was done with the RDP classifier at a confidence level of 80% with *Aquifex pyrophilus* as the outgroup. The tree was constructed in iTOL (7). A high resolution figure is available at: http://itol.embl.de/shared/katharina

Legend Fig. S2

unclassified Bacteria
Proteobacteria
Nitrospira
Gemmatimonadetes
Bacteroidetes
Verrucomicrobia
Chloroflexi
Acidobacteria
Actinobacteria
Armatimonadetes
Planctomycetes
Firmicutes
Candidate division *OD1*

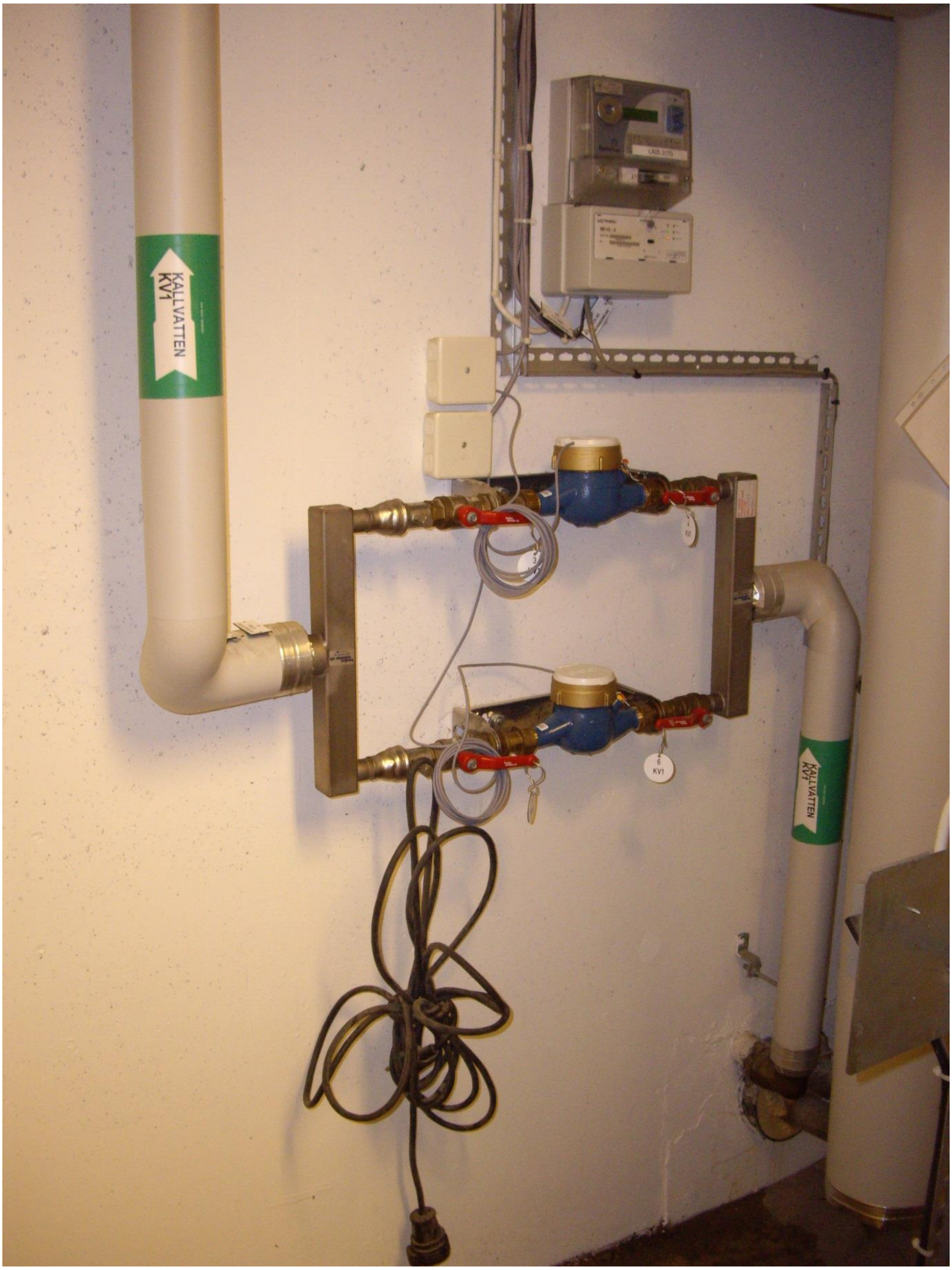

Fig. S3

Parallel installed water meters where biofilms samples of WM 1 and WM 2 were obtained. The water flows through both water meters at the same time. The white arrows on the green background, located on the white pipes indicate the direction of the water flow.

Table S1 - Sequences for the 20 most abundant OTUs for the V1-V2 and V3 region of the bacterial 16S rRNA gene
The sequence for each OTU can be found under the table listed by the 454 identifier in the last column.

V1-V2 region

Phylum	Class	Order	Family	Genus	WM 1 - F	WM 2 - F	WM 3 - F	WM 4 - F	P1 - F	P2 - F	454 identifier
Actinobacteria	*Actinobacteria*	*Acidimicrobiales*			0	0	0	0	1,875	0	G9PMCDE02FUY1Q
Actinobacteria	*Actinobacteria*	*Actinomycetales*	*Mycobacteriaceae*	*Mycobacterium*	118	69	81	4	3,369	859	G9PMCDE02FLZ96
Actinobacteria	*Actinobacteria*	*Actinomycetales*	*Nocardiaceae*	*Nocardia*	2	3	2	3	5,050	2	G9PMCDE02GKHU6
Gemmatimonadetes	*Gemmatimonadetes*	*Gemmatimonadales*	*Gemmatimonadaceae*	*Gemmatimonas*	523	318	6	7	87	141	G9PMCDE01BBNFD
Nitrospira	*Nitrospira*	*Nitrospirales*	*Nitrospiraceae*	*Nitrospira*	196	146	117	2,852	2	137	G9PMCDE01D4KP9
Proteobacteria	*Alphaproteobacteria*	*Rhizobiales*	*Hyphomicrobiaceae*	*Hyphomicrobium*	1,401	1,715	580	751	1,701	664	G9PMCDE01A2A8S
Proteobacteria	*Alphaproteobacteria*	*Rhizobiales*	*Hyphomicrobiaceae*	*Pedomicrobium*	488	363	8	1,949	78	553	G9PMCDE02JO8D8
Proteobacteria	*Alphaproteobacteria*	*Rhizobiales*	*Hyphomicrobiaceae*		1,144	2,332	5,171	102	543	380	G9PMCDE01BMNYR
Proteobacteria	*Alphaproteobacteria*	*Rhizobiales*			3,723	2,263	59	44	321	423	G9PMCDE01EH9I9
Proteobacteria	*Alphaproteobacteria*	*Rhizobiales*			378	336	55	109	146	205	G9PMCDE02H6QCL
Proteobacteria	*Alphaproteobacteria*	*Rhizobiales*			570	312	89	76	99	71	G9PMCDE01AK6V9
Proteobacteria	*Alphaproteobacteria*	*Sphingomonadales*	*Sphingomonadaceae*		5,952	9,578	10,689	149	581	604	G9PMCDE01D9E8L
Proteobacteria	*Alphaproteobacteria*	*Sphingomonadales*	*Sphingomonadaceae*		935	617	17	48	160	369	G9PMCDE02JBXWJ
Proteobacteria	*Alphaproteobacteria*				1,084	807	12	715	265	623	G9PMCDE01AER9F
Proteobacteria	*Betaproteobacteria*				753	579	10	334	98	1,466	G9PMCDE02G5DQD
Proteobacteria	*Betaproteobacteria*	*Burkholderiales*	*Burkholderiales_incertae_sedis*	*Methylibium*	85	27	7	33	24	1,434	G9PMCDE01ADPBM
Proteobacteria	*Deltaproteobacteria*	*Desulfovibrionales*	*Desulfovibrionaceae*	*Desulfovibrio*	1	1	0	0	1	2,470	G9PMCDE02GUPUE
Proteobacteria	*Epsilonproteobacteria*	*Campylobacterales*	*Helicobacteraceae*	*Sulfuricurvum*	1	0	0	0	0	1,476	G9PMCDE01DS1Y1
Proteobacteria	*Gammaproteobacteria*				0	0	2	0	8,621	0	G9PMCDE02GSO2W
Proteobacteria					1,542	1,258	3,490	56	106	13	G9PMCDE01DYPOI
				Percentage of total sequences	71%	78%	77%	27%	87%	45%	

V3 region

Phylum	Class	Order	Family	Genus	WM 1 - R	WM 2 - R	WM 3 - R	WM 4 - R	P1 - R	P2 - R	454 identifier
Actinobacteria	*Actinobacteria*	*Acidimicrobiales*			0	3	0	2	1,630	1	G9PMCDE02HNUTA
Actinobacteria	*Actinobacteria*	*Actinomycetales*			146	106	103	101	8,445	919	G9PMCDE02JQV4U
Nitrospira	*Nitrospira*	*Nitrospirales*	*Nitrospiraceae*	*Nitrospira*	168	157	119	2,800	5	94	G9PMCDE02GR3N0
Proteobacteria	*Alphaproteobacteria*	*Caulobacterales*	*Caulobacteraceae*		998	667	6	677	193	523	G9PMCDE02F4VF2
Proteobacteria	*Alphaproteobacteria*	*Rhizobiales*	*Hyphomicrobiaceae*		7,398	6,937	5,832	4,007	2,645	2,530	G9PMCDE01EGEG4
Proteobacteria	*Alphaproteobacteria*	*Rhizobiales*			365	251	10	145	247	432	G9PMCDE02GIDE9
Proteobacteria	*Alphaproteobacteria*	*Sphingomonadales*	*Sphingomonadaceae*		6,913	10,667	10,808	254	614	1,166	G9PMCDE02IR4IB
Proteobacteria	*Alphaproteobacteria*				257	217	75	1,175	55	400	G9PMCDE02IEKYC
Proteobacteria	*Alphaproteobacteria*				253	206	53	1,204	104	235	G9PMCDE02I0N9S
Proteobacteria	*Betaproteobacteria*	*Burkholderiales*	*Comamonadaceae*		619	331	53	46	53	1,015	G9PMCDE02HIN1O
Proteobacteria	*Betaproteobacteria*	*Burkholderiales*	*Oxalobacteraceae*	*Herminiimonas*	562	420	21	38	375	409	G9PMCDE02JU99X
Proteobacteria	*Betaproteobacteria*	*Burkholderiales*			131	30	16	62	49	1,800	G9PMCDE02IM4RT
Proteobacteria	*Betaproteobacteria*	*Rhodocyclales*	*Rhodocyclaceae*		259	170	52	245	102	629	G9PMCDE02ILVSY
Proteobacteria	*Betaproteobacteria*				840	639	10	369	92	1,599	G9PMCDE01BYOG5
Proteobacteria	*Deltaproteobacteria*	*Desulfovibrionales*	*Desulfovibrionaceae*	*Desulfovibrio*	2	0	0	0	0	2,779	G9PMCDE02I385K
Proteobacteria	*Epsilonproteobacteria*	*Campylobacterales*	*Helicobacteraceae*	*Sulfuricurvum*	4	2	0	0	0	1,854	G9PMCDE02HJK11
Proteobacteria	*Gammaproteobacteria*				0	2	12	0	9,805	0	G9PMCDE02HIBGD
Proteobacteria					1,588	1,225	3,596	38	65	2	G9PMCDE02G3AQH
					439	298	21	905	87	141	G9PMCDE02HZZG9
					0	0	0	1,820	0	0	G9PMCDE01EBZ4Q
				Percentage of total sequences	79%	84%	79%	52%	93%	62%	

Sequences from OTUs in Table S1

>G9PMCDE02FUY1Q
GACGAACGTTGGCGGCGTGCCTAACACATGCAAGTCGAGCGAGGTCCATCCAGT
GGTAACACTGGTGAAGACCTAGCGGCGAACGGGTGAGTAACACGTGAGAAACCT
GCCCCGAAGACTGGGATAACTCTCCGAAAGGAGAGCTAATACCAGATGCCCTCA
CCGGGCCGCATGACTTGGTGAGGAAATGGATTCCGCTTCGGGAGGGTCTCGCGGC
CTATCAGCTTGTTGGTGAGGTAACGGCTCACCAAGGCTTCGACGGGTAGCTGGTC
TGAGAGGATGATCAGCCACACTGGGACTGAGACACGGCCCAGACTCCTACGGGA
GGCA

>G9PMCDE02FLZ96
GACGAACGCTGGCGGCGTGTTTAACACATGCAAGTCGAACGGAAAGGCCCTTCG
GGGTACTCGAGTGGCGAACGGGTGAGTAACACGTGGGTGATCTGCCCTGCACTTC
GGGATAAGCCTGGGAAACTGGGTCTAATACCGGATATACCTCTTGGATCGCATGG
TCTTTGGGGGAAAGCTTTTGCGGTGTGGGATGGGCCCGCGGCCTATCAGCTTGTT
GGTGGGGTGATGGCCTACCAAGGCGTCGACGGGTAGCCGGCCTGAGAGGGTGTC
CGGCCACACTGGGACTGAGATACGGCCCAGACTCCTACGGGAGGCAGCAGTGGG
GAA

>G9PMCDE02GKHU6
GACGAACGCTAGCGGCGTGCTTAACACATGCAAGTCGAGCGGTAAGGCCCTTCG
GGGTACACGAGCGGCGAACGGGTGAGTAACACGTGGGTGATCTGCCTTGCACTTC
GGGATAAGCCTGGGAAACCGGGCCTAATACCGGATATGACCACGAGATGCATGT
CTTGTGGTGGAAAGATTTATCGGTGCGAGATGGGCCCGCGGCCTATCAGCTTGTT
GGTGAGGTAACGGCTCACCAAGGCGACGACGGGTAGCCGACCTGAGAGGGTGAC
CGGCCACACTGGGACTGAGACACGGCCCAGACTCCTACGGGAGGCAGCAGTGGG
GAA

>G9PMCDE01BBNFD
GACGAACGCTGGCGGCGTGCTTAACACATGCAGTCGCACGGCCCGCAAGGGCAG
TGGCGGACGGGTGCGTAACACGTGAAGAATGTACCCATTGGCGGGGGATAGCCG
GCCCAACGGCCGGGTAATACCGCATACGCAGCGCTGGGGGCATCCCCGGGGTTG
GAAAGCCGTAAGGCACCGATGGAGCACTTCGCGGCCTATCAGCTAGTTGGTGTGG
TCATGGCGCACCAAGGCGACGACGGGTAGCTGGTCTGAGAGGATGATCAGCCAC
ATTGGGACTGAGACACGGCCCAGACTCCTACGGGAGGCAGCAGTGGGGAATATT
GCGCA

>G9PMCDE01D4KP9
AATGAACGCTGGCGGCGCGCCTAATACATGCAAGTCGAGCGAGAAGACGTAGCA
ATACGTTTGTAAAGCGGCGAACGGGTGAGGAATACATGGGTAACCTACCCTCGA
GTGGGGAATAACTAGCCGAAAGGTTAGCTAATACCGCGTACGCTCCTTGGACTTC
GGTTCAGGGAGGAAAGCGATACCGTGGGTATCGCGCTCTTGGATGGGCTCATGTC
CTATCAGCTTGTTGGTGAGGTAACGGCTCACCAAGGCTTCGACGGGTAGCTGGTC
TGAGAGGACGATCAGCCACACTGGCACTGCGACACGGGCCAG

>G9PMCDE01A2A8S
AACGAACGCTGGCGGCAGGCCTAACACATGCAAGTCGAACGCCCTAGCAATAGG
GAGTGGCAGACGGGTGAGTAACACGTGGGAACCTTCCCTATAGTACGGAATAGC
CCAGGGAAACTTGGAGTAATACCGTATACGCCCGAAAGGGGAAAGATTTATCGC
TATAGGATGGGCCCGCGTAGGATTAGCTAGTTGGTGAGGTAATGGCTCACCAAGG
CGACGATCCTTAGCTGGTTTGAGAGAATGACCAGCCACACTGGGACTGAGACAC
GGCCCAGACTCCTACGGGAGGCAGCAGTGGGGAATATTGGACAATGGGCGCAAG
CCTGA

>G9PMCDE02JO8D8
AACGAACGCTGGCGGCAGGCCTAACACATGCAAGTCGAACGCTCGTAGCAATAC
GAGAGTGGCAGACGGGTGAGTAACACGTGGGAACCTTCCCTATGGTTCGGAATA
GCTCAGGGAAACTTGGGGTAATACCGGATAAGCCCTTCGGGGGAAAGATTTATC
GCCATTGGATGGGCCCGCGTCGGATTAGCTAGTTGGTGAGGTAACGGCTCACCAA
GGCGACGATCCGTAGCTGGTCTGAGAGGATGATCAGCCACACTGGGACTGAGAC
ACGGCCCAGACTCCTACGGGAGGCAGCAG

>G9PMCDE01BMNYR
AACGAACGCTAGCGGCAGGCCTAACACATGCAAGTCGAACGCTCCGCAAGGGGA
GTGGCAGACGGGTGAGTAACACGTGGGAATCTTCCTATTGGTATGGAATAGCTCA
GGGAAACTTGGGGTAATACCGTATAAGCCCGAGAGGGGAAAGATTTATCGCCAA
TAGATGAGCCCGCGTCTGATTAGCTAGTTGGTGAGGTAATGGCTCACCAAGGCGA
CGATCAGTAGCTGGTCTGAGAGGATGATCAGCCACACTGGGACTGAGACACGGC
CCAGACTCCTACGGGAGGCAGCAGTGGGGAATATTGGACAATGGGCGCAAGCCT
GATC

>G9PMCDE01EH9I9
AACGAACGCTGGCGGCAGGCCTAACACATGCAAGTCGAGCGCCCTAGCAATGGG
GAGCGGCAGACGGGTGAGTAACGCGTGGGTATCTACCTAGTGGTGCGGAATAGC
CCAGGGAAACTTGGAGTAATACCGCATAAGCCCGAAAGGGGAAAGATTTATCGC
CATTAGATGAGCCCGCGTTGGATTAGCTAGTTGGTGGGGTAATGGCCCACCAAGG
CGACGATCCATAGCTGGTCTGAGAGGATGATCAGCCACACTGGGACTGAGACAC
GGCCCAGACTCCTACGGGAGGCAGCAGTGGGGAATATTGGACAATGGGCG

>G9PMCDE02H6QCL
GACGAACGCTGGCGGCAGGCTTAACACATGCAAGTCGAGCGCCCCGCAAGGGGA
GCGGCAGACGGGTGAGTAACGCGTGGGAACGTGCCTTTCGGTTCGGAACAACTG
AGGGAAACTTCAGCTAAAACCGGATACGCCCTTCGGGGAAAGTTTACGCCGAAA
GATCGGCCCGCGTCAGATTAGGTAGTTGGTGGGGTAATGGCCTACCAAGCCTACG
ATCTGTAGCTGTTCTGAGAGGAAGATCAGCCACACTGGGACTGAGACACGGCCC
AGACTCCTACGGGAGGCAGCAGTGGGGAATATTGGACAATGGGCGCAAGCCTGA
TCCAG

>G9PMCDE01AK6V9
AACGAACGCTGGCGGCAGGCCTAACACATGCAAGTCGAGCGCTGTAGCAATACA
GAGCGGCAGACGGGTGGGTAACGCGTGGGAATTTACCTGATGGTACGGAATAGC
CCAGGGAAACTTGGGGTAATACCGTATAAGCCCGAGAGGGGAAAGATTTATCGC
CATCAGATGAGCCCGCGTTGGATTAGCTAGTTGGTGAGGTAACGGCTCACCAAGG
CGACGATCCATAGCTGGTCTGAGAGGATGACCAGCCACACTGGGACTGAGACAC
GGCCCAGACTCCTACGGGAGGCAGCAGTGGGGAATCTTGGACAATGGGCGCAAG
CCTGA

>G9PMCDE01D9E8L
AACGAACGCTGGCGGCATGCCTAACACATGCAAGTCGAACGAGACCTTCGGGTC
TAGTGGCGCACGGGTGCGTAACGCGTGGGAATCTGCCCTTGGGTTCGGAATAACT
TCGGGAAACTGAAGCTAATACCGGATGATGACGTAAGTCCAAAGATTTATCGCCC
AAGGATGAGCCCGCGTAGGATTAGCTAGTTGGTGAGGTAAAGGCTCACCAAGGC
GACGAT

>G9PMCDE02JBXWJ
AACGAACGCTGGCGGCATGCTTAACACATGCAAGTCGAACGAGACCTTCGGGTCT
AGTGGCGCACGGGTGCGTAACGCGTGGGAATCTGCCCTTAGGTACGGAATAACTC
AGAGAAATTTGTGCTAATACCGTATGATGTCGAAAGACCAAAGATTTATCGCCTA
AGGATGAGCCCGCGTAAGATTAGCTTGTTGGTGAGGTAAAAGCTCACCAAGGCG
ACGATCTTTAGCTGGTCTGAGAGGATGATCAGCCACACTGGGACTGAGACACGGC
CCAGACTCCTACGGGAGGCAGCAGTGGGGAATATTGGACAATGGGCGAAAGCCT
GA

>G9PMCDE01AER9F
AACGAACGCTGGCGGCAGGCCTAACACATGCAAGTCGAGCGCCCCGCAAGGGGA
GCGGCGGACGGGTGAGTAACACGTGGGAATTTTCCTCAAGGTACGGAACAACTC
AGGGAAACTTGGGCTAATACCGTATGATGCAGAGATGCCAAAGATTTATCGCCTT
GGGATAAGCCCGCGTCAGATTAGGTAGTTGGTGGGGTAA

>G9PMCDE02G5DQD
ATTGAACGCTGGCGGCATGCCTTACACATGCAAGTCGAACGCGAAAGCGGGGCA
ACCCGCAAGTAGAGTGGCGAACGGGTGAGTAATACATCGGAACATATCCAGTAG
CGGGGGATAACCTATCGAAAGATGGGCTAATACCGCATACGATCTACGGATGAA
AGCAGGGGACCGCAAGGCCTTGCACTATTGGAGTGGCCGATGTCGGATTAGCTA
GTTGGTGAGGTAAAGGCTTACCAAGGCGACGATCCGTAGCTGGTCTGAGAGGAC
GATCAGCCACACTGGAACTGAGACACGGTCCAGACTCCTACGGGAGGCAGCAGT
GGGGAA

>G9PMCDE01ADPBM
ATTGAACGCTGGCGGCATGCCTTACACATGCAAGTCGAACGGCAGCACGGGAGC
AATCCTGGTGGCGAGTGGCGAACGGGTGAGTAATACATCGGAACGTGCCCAGTT
GTGGGGGATAGCCCGGCGAAAGCCGGATTAATACCGCATACGACCTACGGGTGA
AAGCGGGGGATCGCAAGACCTCGCGCTATTGGAGCGGC

>G9PMCDE02GUPUE
ATTGAACGCTGGAGGCGTGCTTAACACATGCAAGTCGTGCGAGAAAGGGACTTC
GGTCCTGAGTAGAGCGGCGCACGGGTGAGTAACGCGTGGGTAATCTCCCTTGGTG
ACTGGGATAACAGCGGGAAACTGCTGCTAATACCGGATATATTTCATGTTTAACT
TCATGGGGAAAAGGTGGCCTCTGCTTGCAAGCTACCACACCGAGATGAGCCCGC
GTCTCATTAGCTAGTTGGTAAGGTAATGGCTTACCAAGGCTACGATGAGTAGCTG
GTCTGAGAGGATGACCAGCCACACTGGGACTGGAACACGGCCCAGACTCCTACG
GGA

>G9PMCDE01DS1Y1
AGTGAACGCTGGCGGCGTGCCTAACACATGCAAGTCGAACGATGATTGCGGAGC
TTGCTCTGCATGATTAGTGGCGCACGGGTGAGTATACCATAGATAATGTACCCCT
TAGTTCGGGATAGCCACTGGAAACGGTGATTAATACCGGATACTCCTTCTTGTCT
CAAGGCAAGTCGGGAAAGTTTTTTCGCTAAGGGATCAGTCTATGTCCTATCAGCT
AGTTGGTGAGGTAATGGCTCACCAAGGCTATGACGGGTATCTGGTTTGAGAGGAT
GATCAGACACACT

>G9PMCDE02GSO2W
ATTGAGCGCTGGCGGCATGCCTAACACATGCAAGTCGAACGGTAGCAGGTCCTTC
GGGATGCTGACGAGTGGCGGACGGGTGCGTAACGCGTGGGAATCTGCCCAATAG
TGGGGGATAACCCGGGGAAACCCGGGCTAATACCGCATACTCCCTACGGGGGAA
AGCGGGGGACCGCAAGGCCTCGCGCTATTGGATGAGCCCGCGTCCGATTAGCTTG
TTGGTGGGGTAAAAGCCTACCAAGGCGACGATCGGTAGCTGGTCTGAGAGGACG
ACCAGCCACACTGGGACTGAGACACGGCCCAGACTCCTACGGGAGGCAGCAGTG
GGGA

>G9PMCDE01DYPOI
AACGTACGCTGGCGGCACGCCTAACACATGCAAGTCGAACGCAGTAGCAATACT
GAGTGGCAGACGGGTGAGTAACATGTGGGAATCTGCCTTATGGTTCAGGACAAC
ATTGGGAAACCGATGCTAATACTGGATAAGCCCTTACGGGGAAAGTTTTAATGCC
ATAAGATGAGCCCGCATTTGATTAGTTAGTTGGTGAGGTAATGGCTCACCAAGAC
AATGATCAATAGCTGATCTGAGAGGATGATCAGCCACACTGGGACTGAGACACG
GCCCAGACTCCTACGGGAGGCAGCAGTGG

>G9PMCDE02HNUTA
TCGGGAGGGTCTCGCGGCCTATCAGCTTGTTGGTGAGGTAACGGCTCACCAAGGC
TTCGACGGGTAGCTGGTCTGAGAGGATGATCAGCCACACTGGGACTGAGACACG
GCCCAGACTCCTACGGGAGGCAGCAGTGGGGAATCTTGCGCAATGGGCGAAAGC
CTGACGCAGCAACGCCGCGTGGGGGATGAAGGCTCTCGGGTTGTAAACCCCTTTC
AGCAGGGAAGATTATGACGGTACCTGCAGAAGAAGCCCCGGCCAACTACGT

>G9PMCDE02JQV4U
CCAAGGCGACGACGGGTAGCCGGCCTGAGAGGGTGACCGGCCACACTGGGACTG
AGATACGGCCCAGACTCCTACGGGAGGCAGCAGTGGGGAATATTGCACAATGGG
CGCAAGCCTGATGCAGCGACGCCGCGTGAGGGATGACGGCCTTCGGGTTGTAAA
CCTCTTTCAGCAGGGACGAAGCGCAAGTGACGGTACCTGTAGAAGAAGGACCGG
CCAACTACGT

>G9PMCDE02GR3N0
CGTGGGTATCGCGCTCTTGGTTGGGCTCATGTCCTATCAGCTTGTTGGTGAGGTAA
CGGCTCACCAAGGCTTCGACGGGTAGCTGGTCTGAGAGGACGATCAGCCACACT
GGCACTGCGACACGGGCCAGACTCCTACGGGAGGCAGCAGTAAGGAATATTGCG
CAATGGGCGAAAGCCTGACGCAGCGACGCCGCGTGGGGGATGAAGGTCTTCGGA
TTGTAAACCCCTTTCGGGAGGGAAGATGGAGTGGGGTAACCCATTCGGACGGTAC
CTCCAGAAGCAGCCACGGCTAACTTCGT

>G9PMCDE02F4VF2
ATCTGTAGCTGGTCTGAGAGGACGATCAGCCACATTGGGACTGAGACACGGCCC
AAACTCCTACGGGAGGCAGCAGTGGGGAATCTTGCGCAATGGGCGAAAGCCTGA
CGCAGCCATGCCGCGTGAATGATGAAGGTCTTAGGATTGTAAAGTTCTTTCGCCT
GTGACGATGATGACGGTAACAGGAGAAGAAGCCCCGGCTAACTTCGT

>G9PMCDE01EGEG4
TATCAGTAGCTGGTCTGAGAGGATGATCAGCCACACTGGGACTGAGACACGGCC
CAGACTCCTACGGGAGGCAGCAGTGGGGAATATTGGACAATGGGCGCAAGCCTG
ATCCAGCCATGCCGCGTGAGTGACGAAGGTCTTCGGATTGTAAAGCTCTTTTGGC
GGGGACGATAATGACGGTACCCGCAGAATAAGCCCCGGCTAACTTCGT

>G9PMCDE02GIDE9
CTACCAAGGCGACGATCCATAGCTGGTCTGAGAGGACGACCAGCCACACTGGGA
CTGAGACACGGCCCAGACTCCTACGGGAGGCAGCAGTGGGGAATCTTGGACAAT
GGGCGCAAGCCTGATCCAGCCATGCCGCGTGAGTGATGAAGGCCCTAGGGTTGT
AAAACTCTTTCGTGGGGGACGATAATGACGGTACCCCAAGAAGAAGCTCCGGCT
AACTTCGT

>G9PMCDE02IR4IB
CCAAGGATGAGCCCGCGTAGGATTAGCTAGTTGGTGAGGTAAAGGCTCACCAAG
GCGACGATCCTTAGCTGGTCTGAGAGGATGATCAGCCACACTGGGACTGAGACA
CGGCCCAGACTCCTACGGGAGGCAGCAGTGGGGAATATTGGACAATGGGCGAAA
GCCTGATCCAGCAATGCCGCGTGAGTGATGAAGGCCTTAGGGTTGTAAAGCTCTT
TTACCCGGGATGATAATGACAGTACCGGGAGAATAAGCTCCGGCTAACTCCGT

>G9PMCDE02IEKYC
TAGCTGGTCTGAGAGGATGATCAGCCACACTGGGACTGAGACACGGCCCAGACT
CCTACGGGAGGCAGCAGTGGGGAGTATTGGACAATGGGGGCAACCCTGATCCAG
CAATGCCGCGTGAGTGATGAAGGCCTTAGGGTTGTAAAGCTCTTTCGGCGGGGAA
GATGATGACGGTACCCGCAGAAGAAGCCCCGGCTAACTTCGT

>G9PMCDE02I0N9S
CTGTTCTGAGAGGAAGATCAGCCACACTGGGACTGAGACACGGCCCAGACTCCT
ACGGGAGGCAGCAGTGGGGAATATTGGACAATGGGCGCAAGCCTGATCCAGCCA
TGCCGCGTGAGTGATGAAGGCCTTAGGGTTGTAAAGCTCTTTTACCAGGGAAGAT
AATGACGGTACCTGGAGAATAAGCCCCGGCTAACTTCGT

>G9PMCDE02HIN1O
CACCAAGGCAACGATCTGTAGCTGGTCTGAGAGGACGACCAGCCACACTGGGAC
TGAGACACGGCCCAGACTCCTACGGGAGGCAGCAGTGGGGAATTTTGGACAATG
GGCGCAAGCCTGATCCAGCAATGCCGCGTGCAGGACGAAGGCCTTCGGGTTGTA
AACTGCTTTTGTACGGAACGAAACGGTCCTTTCTAATACAGGGGGCTAATGACGG
TACCGTAAGAATAAGCACCGGCTAACTACGT

>G9PMCDE02JU99X
GCTGGTCTGAGAGGACGACCAGCCACACTGGAACTGAGACACGGTCCAGACTCC
TACGGGAGGCAGCAGTGGGGAATTTTGGACAATGGGCGCAAGCCTGATCCAGCA
ATGCCGCGTGAGTGAAGAAGGCCTTCGGGTTGTAAAGCTCTTTTGTCAGGGAAGA
AACGGTGGACTCTAATACAGATCACTAATGACGGTACCTGAAGAATAAGCACCG
GCTAACTACGT

>G9PMCDE02IM4RT
GTCTGAGAGGACGACCAGCCACACTGGGACTGAGACACGGCCCAGACTCCTACG
GGAGGCAGCAGTGGGGAATTTTGGACAATGGGCGCAAGCCTGATCCAGCCATGC
CGCGTGCGGGAAGAAGGCCTTCGGGTTGTAAACCGCTTTTGTCAGGGAAGAAAC
GGTCTGGGCTAATACCTCGGACTAATGACGGTACCTGAAGAATAAGCACCGGCTA
ACTACGT

>G9PMCDE02ILVSY
GGCGACGATCCGTAGCTGGTCTGAGAGGATGATCAGCCACACTGGGACTGAGAC
ACGGCCCAGACTCCTACGGGAGGCAGCAGTGGGGAATTTTGGACAATGGGGGCA
ACCCTGATCCAGCCATGCCGCGTGAGTGAAGAAGGCCTTCGGGTTGTAAAGCTCT
TTCGGACGGAACGAAACGGTTAGCGTGAATATCGCTGACTAATGACGGTACCGTA
AGAAGAAGCACCGGCTAACTACGT

>G9PMCDE01BYOG5
CACACTGGAACTGAGACACGGTCCAGACTCCTACGGGAGGCAGCAGTGGGGAAT
TTTGGACAATGGGCGCAAGCCTGATCCAGCCATGCCGCGTGAGTGAAGAAGGCC
TTCGGGTTGTAAAGCTCTTTCGGTGAAGACGAAACGGTTAGCGCTAATAACGCTG
ACTAATGACGGTACTCACAGAAGAAGCACCGGCTAACTACGT

>G9PMCDE02I385K
GGGTAATGGCCTACCAAGGCTACGATGAGTAGCTGGTCTGAGAGGATGACCAGC
CACACTGGGACTGGAACACGGCCCAGACTCCTACGGGAGGCAGCAGTGGGGAAT
ATTGCGCAATGGGCGAAAGCCTGACGCAGCGACGCCTCGTGAGGGATGAAGGTC
TTCGGATCGTAAACCTCTGTCAAGAGGGAAGAAACCCTTGGATTCGAATAGGGTC
CTTGGCTGACGGTACCTCAAAAGGAAGCACCGGCTAACTCCGT

>G9PMCDE02HJK11
AATACCAGATACTCCTTCTTGTCTCAAGGCAAGTCGGGAAAGTTTTTCGCTAAG
GGATCAGTCTATGTCCTATCAGCTAGTTGGTGAGGTAATGGCTCACCAAGGCTAT
GACGGGTATCTGGTTTGAGAGGATGATCAGACACACTGGAACTGAGACACGGTC
CAGACTCCTACGGGAGGCAGCAGTGAGGAATATTGCACAATGGAGGAAACTCTG
ATGCAGCAACGCCGCGTGGAGGATGACGCATTTCGGTGTGTAAACTCCTTTTATT
AGGGAAGATAATGACGGTACCTAATGAATAAGCACCGGCTAACTCCGT

>G9PMCDE02HIBGD
TACGGGGGAAAGCGGGGGACCGCAAGGCCTCGCGCTATTGGATGAGCCCGCGTC
CGATTAGCTTGTTGGTGGGGTAAAAGCCTACCAAGGCGACGATCGGTAGCTGGTC
TGAGAGGACGACCAGCCACACTGGGACTGAGACACGGCCCAGACTCCTACGGGA
GGCAGCAGTGGGGAATATTGGACAATGGGGGAAACCCTGATCCAGCAATGCCGC
GTGTGTGAAGAAGGCCTGCGGGTTGTAAAGCACTTTCGGCAGGAAAGAAAAGCC
ATGCGCTAATACCGCATGACCTTGACGTTACCTGCAAAAGAAGCACCGGCTAACT
CCGT

>G9PMCDE02G3AQH
AGTACAACATTGGGAAACCGATGCTAATACTGGATAAGCCCTTACGGGGAAAGT
TTTAATGCCATAAGATGAGCCCGCATTTGATTAGTTAGTTGGTGAGGTAATGGCT
CACCAAGACAATGATCAATAGCTGATCTGAGAGGATGATCAGCCACACTGGGAC
TGAGACACGGCCCAGACTCCTACGGGAGGCAGCAGTGGGGAATCTTGCACAATG
GAGGAAACTCTGATGCAGCGATGCCGCGTGAGTGAAGAAGGCCTTTGGGTTGTA
AAGCTCTTTTGTAGGGGAAGATAATGACTGTACCCTAAGAATAAGGTCCGGCTAA
CTTCGT

>G9PMCDE02HZZG9
GACGGGTAGCTGGTCTGAGAGGATGATCAGCCACATTGGGACTGAGACACGGCC
CAGACTCCTACGGGAGGCAGCAGTGGGGAATATTGCGCAATGGGCGCAAGCCTG
ACGCAGCGACGCCGCGTGCGGGATGACGGCCTTCGGGTTGTAAACCGCTGTCGG
GAGGGACGAATTGATGACGGTACCTCCAAAGGAAGCACCGGCTAACTCTGT

>G9PMCDE01EBZ4Q
AGCTGGTCTGAGAGGATGATCAGCCAGACTGGGACTGCGAACGGCCCAGACTCC
TACGGGAGGCAGCAGTTAGGAATTTTGGGCAATGGGCGAAAGCCTGACCCAGCA
ACGCCGCGTGGGTGATGAAGGCCCTCGGGTCGTAAAGCCCTTTTCCCAGGGAAGA
TAGTGACGGTACCTGGGGAATCAGCCCCGGCTAACTACGT

REFERENCES

1. Bengtsson, J., K.M. Eriksson, M. Hartmann, Z. Wang, B.D. Shenoy, G.A. Grelet, K. Abarenkov, A. Petri, M.A. Rosenblad, and R.H. Nilsson. 2011. Metaxa: a software tool for automated detection and discrimination among ribosomal small subunit (12S/16S/18S) sequences of archaea, bacteria, eukaryotes, mitochondria, and chloroplasts in metagenomes and environmental sequencing datasets. Antonie Van Leeuwenhoek 100:471-475.

2. DeSantis, T.Z., Jr., P. Hugenholtz, K. Keller, E.L. Brodie, N. Larsen, Y.M. Piceno, R. Phan, and G.L. Andersen. 2006. NAST: a multiple sequence alignment server for comparative analysis of 16S rRNA genes. Nucleic Acids Res. 34:W394-W399.

3. Edgar, R.C. 2010. Search and clustering orders of magnitude faster than BLAST. Bioinformatics 26:2460-2461.

4. Edgar, R.C., B.J. Haas, J.C. Clemente, C. Quince, and R. Knight. 2011. UCHIME improves sensitivity and speed of chimera detection. Bioinformatics 27:2194-2200.

5. Gilbert, J.A., D. Field, P. Swift, L. Newbold, A. Oliver, T. Smyth, P.J. Somerfield, S. Huse, and I. Joint. 2009. The seasonal structure of microbial communities in the Western English Channel. Environ. Microbiol. 11:3132-3139.

6. Hao, X., R. Jiang, and T. Chen. 2011. Clustering 16S rRNA for OTU prediction: a method of unsupervised Bayesian clustering. Bioinformatics 27:611-618.

7. Letunic, I., and P. Bork. 2007. Interactive Tree Of Life (iTOL): an online tool for phylogenetic tree display and annotation. Bioinformatics 23:127-128.

8. Schmieder, R., and R. Edwards. 2011. Quality control and preprocessing of metagenomic datasets. Bioinformatics 27:863-864.

9. Stamatakis, A. 2006. RAxML-VI-HPC: maximum likelihood-based phylogenetic analyses with thousands of taxa and mixed models. Bioinformatics 22:2688-2690.

10. Wang, Q., G.M. Garrity, J.M. Tiedje, and J.R. Cole. 2007. Naive Bayesian classifier for rapid assignment of rRNA sequences into the new bacterial taxonomy. Appl. Environ. Microbiol. 73:5261-5267.
